# Reliable and robust robotic handling of microplates via computer vision and touch feedback

**DOI:** 10.3389/frobt.2024.1462717

**Published:** 2025-01-07

**Authors:** Vincenzo Scamarcio, Jasper Tan, Francesco Stellacci, Josie Hughes

**Affiliations:** ^1^ Supramolecular Nano-Materials and Interfaces Laboratory, Institute of Materials, School of Engineering, École Polytechnique Fédérale de Lausanne (EPFL), Lausanne, Switzerland; ^2^ CREATE Lab, Institute of Mechanical Engineering, School of Engineering, École Polytechnique Fédérale de Lausanne (EPFL), Lausanne, Switzerland

**Keywords:** robot manipulation, automation, computer vision, life science, mobile robotics

## Abstract

Laboratory automation requires reliable and precise handling of microplates, but existing robotic systems often struggle to achieve this, particularly when navigating around the dynamic and variable nature of laboratory environments. This work introduces a novel method integrating simultaneous localization and mapping (SLAM), computer vision, and tactile feedback for the precise and autonomous placement of microplates. Implemented on a bi-manual mobile robot, the method achieves fine-positioning accuracies of ± 1.2 mm and ± 0.4°. The approach was validated through experiments using both mockup and real laboratory instruments, demonstrating at least a 95% success rate across varied conditions and robust performance in a multi-stage protocol. Compared to existing methods, our framework effectively generalizes to different instruments without compromising efficiency. These findings highlight the potential for enhanced robotic manipulation in laboratory automation, paving the way for more reliable and reproducible experimental workflows.

## 1 Introduction

Robotic mobile manipulation platforms are increasingly used for the automation of laboratory sciences as they improve consistency and reliability in experimental data capture while enabling large-scale experiments (Abolhasani and Kumacheva, 2023; Thurow, 2021; Holland and Davies, 2020). However, such mobile manipulators (Ghodsian et al., 2023) often struggle to robustly, precisely, and reliably pick and place labware—typically “well plates,” a fundamental task for many wet laboratory protocols. Performing this task in a laboratory is challenging due to the dynamic nature of the environment, variability in instrument locations (Hvilshøj et al., 2012), and the necessity for robots to work alongside humans for extended periods (Duckworth et al., 2019). Additionally, the precision required is often within the millimeter range (Bostelman et al., 2016; Madsen et al., 2015). In laboratory environments, the positioning of instruments is not always fixed; large, fragile devices may need to be moved for various reasons, such as reconfiguring for different experiments, optimizing workflow efficiency, or performing maintenance. This introduces variability that robots must compensate for when performing tasks such as picking and placing labware. Enhancing the reliability and precision of these robots would expand the range of complex wet laboratory protocols that could be automated.

Mobile manipulators commonly rely on simultaneous localization and mapping (SLAM) (Sánchez-Ibáñez et al., 2021) and predefined maps to reach target locations, yet this approach often lacks the necessary localization accuracy, calling for the integration of additional methods. One popular strategy adopts computer vision-based localization, where a stereo camera detects fiducial markers to estimate the instrument’s pose. This technique has enabled automated cell culture workflows (Lieberherr and Peter, 2021) and has also been applied to automate the synthesis of oxygen-producing catalysts from Martian meteorites (Zhu et al., 2023). Furthermore, it has been shown that a mobile manipulator can interact with different workstations to automate sample preparation for a high-performance liquid chromatography (HPLC) device (Wolf et al., 2024). More complex camera systems have also been used; a dual-handed mobile robot adopted a 3D camera for identifying and handling various types of labware based on object features rather than fiducial markers (Ali et al., 2016). Similarly, a robotic arm paired with a depth camera has been shown to autonomously handle and arrange centrifuge tubes in trays (Nguyen et al., 2024). Also, vision systems have been paired with mobile mini-robots and coupled with static robotic arms to facilitate sample delivery (Laveille et al., 2023; You et al., 2017). However, stereo vision and 3D cameras remain sensitive to light conditions and reflections, which limit their long-term reliability (Kalaitzakis et al., 2021). An alternative localization strategy utilizes touch feedback on a cube to determine multiple bench locations (Burger et al., 2020). When deployed in a laboratory, this method enabled a mobile robot to operate continuously for 6 days to perform catalyst optimization experiments. Although this strategy is less commonly used than vision-based localization, the Cooper Group has consistently validated its robustness, expanding the capabilities of the mobile platform over time (Lunt et al., 2024; Dai et al., 2024). However, this strategy requires adding a cube to the laboratory benches and assumes that instruments remain stationary. To maintain reliability, instruments can be secured to benches, and the system can be recalibrated after any unexpected movement.

The potential for the generalization of visual feedback strategies has also been explored. For instance, Wolf et al. (2023) and Wolf et al. (2022) developed a localization framework integrating fiducial markers and barcodes to store device-specific information. Although these approaches have led to robust applications in some contexts, they often lack generalizability and robustness against instrument movement, highlighting the need for further efforts to achieve universal, reliable robotic localization in dynamic laboratory environments.

**FIGURE 1:**
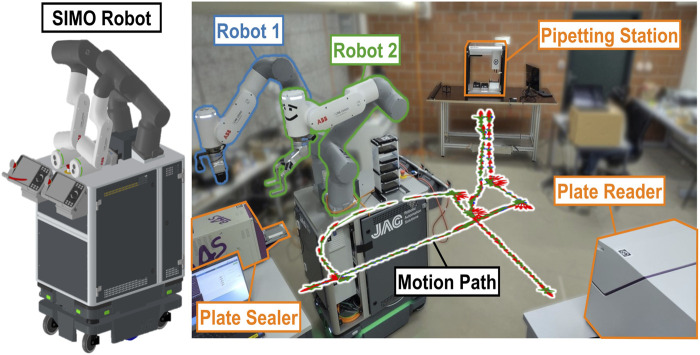
System setup used in this work, consisting of SIMO (left), a bi-manual mobile robot that mounts two robotic arms ( Robot1 and Robot2 ) on a mobile base, and three laboratory instruments (a pipetting station, a plate sealer, and a plate reader). The robot motion path between the three experimental stations is also shown.

In this work, we propose a method that combines visual and tactile detection to precisely estimate the pose of instruments in a laboratory environment. By integrating these methods, we achieve reliable fine detection of the instrument’s pose through tactile feedback while maintaining robustness to unexpected changes using computer vision, thereby leveraging the strengths of both strategies. Additionally, we implemented it on SIMO (smart integrator for manual operations, Figure 1), a bi-manual mobile robot platform. SIMO uses SLAM and VL markers (3D-shaped markers for SLAM) (Wadsten Rex and Klemets, 2019) to localize itself approximately in front of the desired experimental station (defined by a table and one instrument). Then, the robot uses a camera to identify fiducial markers (Benligiray et al., 2019) that are attached to the instruments, thus obtaining their rough pose. Finally, SIMO uses six-point tactile detection on the instrument to obtain its fine pose. We demonstrate the robustness of our method using two mockup instruments by comparing the plate insertion success rate, absolute precision, and mean execution time for different methods. Additionally, we test the generalizability of the concept using three laboratory instruments and perform a “stress test,” where the robot simulates the execution of an experiment five times in a row. We demonstrate that this novel approach can achieve fine positioning ( ±1.2 mm and ±0.4° ) without compromising flexibility or robustness. In this work, we refer to the pick-and-place robustness of the robotic system without implications to the wet laboratory experimental robustness.

In Section 2, we detail our approach and implementation, including the modifications made to the real instruments. Section 3 describes the experimental setup, custom gripper adaptations, and room design. Section 4 presents our findings using both mockup and real instruments. We then conclude with a summary of the obtained results and highlight future research directions in Section 5.

## 2 Methods

### 2.1 Problem statement

Wet laboratory science protocols typically use several bench-top devices and instruments that are spatially distributed in a room. One such protocol is critical micelle concentration (CMC) determination (Mabrouk et al., 2023). This identifies the main physicochemical property of surfactants, which are amphiphilic molecules that decrease surface tension and are key chemicals for disinfection, cleaning, and drug delivery (Falbe 2012; Schramm et al., 2003). This protocol is typically performed by humans; however, this task is work-intensive and prone to errors (Baker, 2016). There is an increasing need for extensive CMC measurements, and thus, a fully automated robotic system is required. To date, no such system exists (Mabrouk et al., 2023).

To automate this task, a robot is required to handle standard microplates (ANSI SLAS 1-2004) with millimeter precision (1−2mm) and transport them between three instruments, namely, a pipetting station, a plate sealer, and a plate reader. This multi-stage process typically takes 1–2 h, depending on the technique (Mabrouk et al., 2023). Additionally, the locations of the instruments may be subject to disturbances and cannot be precisely known in advance.

In this work, we focus on solving the problem of picking and placing microplates between a variety of instruments, where the instruments may be moved or adjusted over time, using a mobile manipulator. This approach is key to enabling many laboratory automation experiments such as CMC.

### 2.2 SIMO robot platform

The system we have developed for CMC and other wet laboratory experiments is a two-arm robot platform, SIMO. The robot is shown in Figure 1; it mounts two GoFa CRB 15000 (ABB, Switzerland) compliant robot arms, namely, Robot 1 and Robot 2 . Robot 1 is equipped with a parallel gripper (2FG7, OnRobot, Denmark) that holds a bespoke, 3D-printed probe, which is used for impedance-based touch localization, and a wrist mounted webcam (c505, Logitech, Switzerland). Robot 2 has a two-finger gripper (RG2, OnRobot, Denmark), which is adapted for gripping standard microplates through the addition of metallic fingers with soft silicone bands.

The dual-arm configuration is beneficial for laboratory automation applications. It mimics human dexterity and bi-manual coordination, enabling robots to handle more complex tasks such as simultaneous manipulation of multiple objects or operating on different parts of an experiment concurrently. The two robotic arms are connected to a robot base (250, MiR, Denmark) via a casing that hosts the arms’ controllers, an onboard PC (NGC-5, Minix, China), and a battery to power the arms. The MiR base uses odometry to estimate its pose with a precision of ±50 mm (Wadsten Rex and Klemets, 2019); SLAM enables the robot to build a map of its surroundings (Figure 7).

Although the developed methods are demonstrated and deployed on this robot, they are potentially generalizable to other mobile manipulators operating in a laboratory environment.

### 2.3 Instrument perception method

Typically, a robotic mobile base can leverage computer vision and SLAM to achieve a precision of ±10 mm . However, this is insufficient for handling microplates. We introduce a generalizable method for any number and type of instruments, which combines vision and touch feedback to accurately pick and place plates.

**FIGURE 2:**
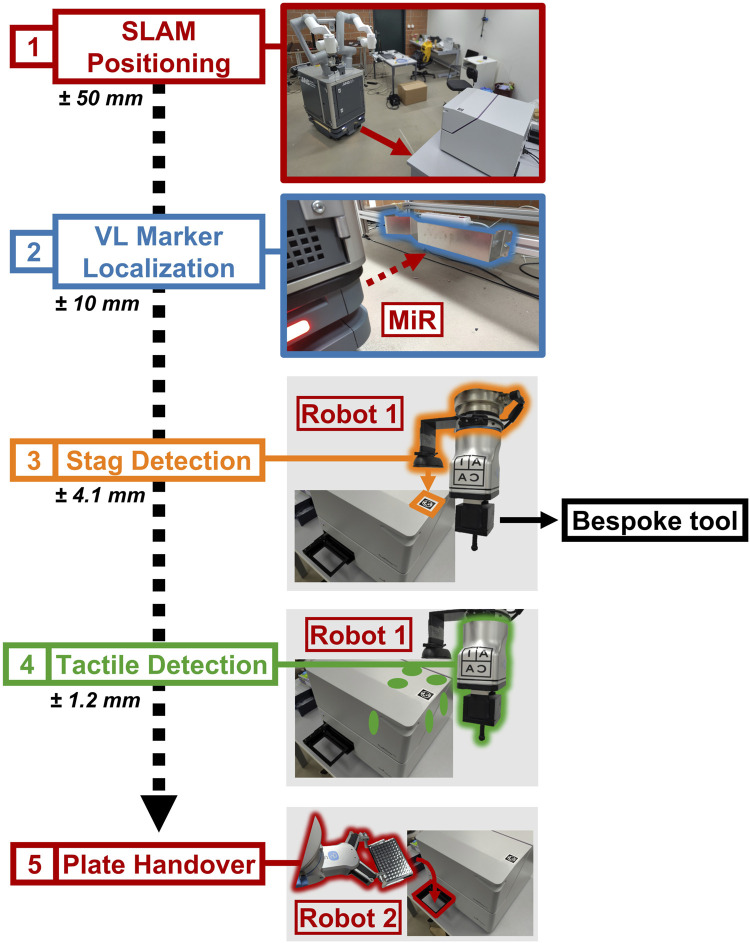
Flow diagram detailing the robot–instrument interaction, highlighting the steps in sequence. SIMO uses SLAM to move to the waypoint, defined for every experimental station (1), and then it localizes the VL Marker, associated with the station (2). Robot1 uses computer vision to estimate the marker’s pose (3), and then it exploits impedance control and the specialized tool to touch the instrument on three faces (six-point touch feedback) (4). Robot2 can perform plate placement after it is informed by Robot1 about the instrument’s pose (5).

Figure 2 details the high-level approach of the method. Using a map that has been previously recorded (Figure 7), SIMO moves to a predefined waypoint for each instrument with a precision of ±50 mm . Using a VL marker, a 3D cut-out with a fixed shape, the LiDAR can obtain a higher accuracy localization in the order of ±10mm (Wadsten Rex and Klemets, 2019). We then assume the robot to be in the rough area where the instrument is; however, its true location can be subject to small disturbances. Additionally, we assume that a fiducial marker is placed in a visible area on the instrument. Using the Robot 1 ’s wrist camera, SIMO estimates the marker’s pose, achieving a measured precision of ±4.1mm . Then, SIMO uses the Robot 1 ’s tactile feedback on the instrument to estimate its corner points and reconstruct its pose with a measured precision of ±1.2mm . Finally, given the location of the plate storage, SIMO can accurately pick and place the plate in the instrument’s handover position (the position where the standard microplates are placed to be processed by the instrument) using Robot 2 .

It should be noted that the VL marker localization step shown in Figure 2 may become redundant if additional steps are implemented to close the loop. Therefore, not all strategies discussed in Sections 2.5 and 4.1 incorporate this step. Nonetheless, because the VL marker step enhances the overall robustness of the method, it is included in the optimal strategy used in Sections 4.2 and 4.3.

Additionally, the precision ranges in Figure 2 are assumed to follow a Gaussian (normal) distribution. However, outliers (although rare) may occur, which the system compensates for through real-time corrections using visual and tactile feedback. By using visual and tactile feedback directly on the instrument without the need to add external cubes, as demonstrated by Burger et al. (2020), SIMO is robust to the instrument’s small, unexpected movements (smaller than a few cm). Additionally, this method facilitates the rapid integration of new instruments, as documented in Section 2.4.5.

### 2.4 Achieving fine positioning

In the following section, we describe the localization strategy once SLAM and VL localization are performed.

Figure 2 shows the approach for precise positioning. Each instrument has an STag marker on a corner. Robot1 ’s wrist camera uses this marker to determine the instrument’s position (3). It then refines this position by touching the instrument at six points with its probe (4). After refining the pose, Robot2 , now informed of the instrument’s exact position, can place the plate in the handover position (5).

#### 2.4.1 Assumptions

**FIGURE 3:**
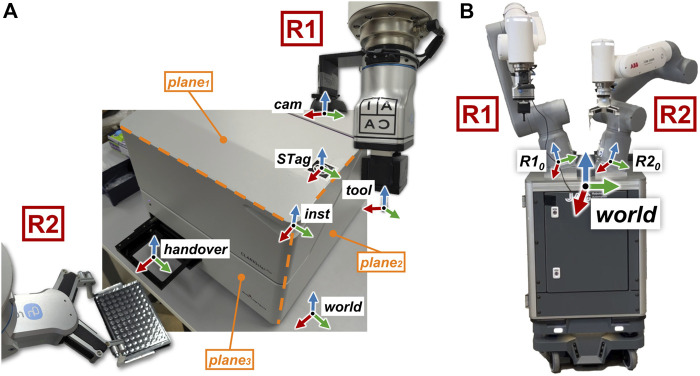
**(A)**: illustration of the main reference frames involved in the robot–instrument interaction, highlighting the touch planes on the instrument. **(B)**: the “world” reference frame is positioned centrally, in between and in front of robotic arms R1 and R2 . The RGB color model represents the axes, with each reference frame labeled accordingly. The labels R1 and R2 designate the two robotic arms.

To develop the algorithms, we make some assumptions about the environment. To describe these assumptions, we introduce several reference frames (see Figure 3).

• Robot 1 (R1) and Robot 2 (R2) share a common reference frame (world) , placed in between and in front of R1 and R2 (Assumption I).

• The instrument must have three perpendicular surfaces ( $plane_{1}$ , $plane_{2}$ , and $plane_{3}$ ), one of them being parallel to the $world_{xy}$ plane (Assumption II).

• The STag marker (STag) must be visible from the camera, and it must be attached to the instrument on a plane that is parallel to the $world_{xy}$ plane (Assumption III).

• The instrument and its handover position must be physically reachable by R1 and R2 (Assumption IV).

It is important to note that, as per Assumption II, the perpendicular surfaces can either be part of the instrument’s original design or added using custom 3D-printed components, as detailed in Section 2.4.5. Based on these assumptions, we can now introduce the different elements of the method.

#### 2.4.2 Visual detection of STag markers

Visual detection is used to estimate the instrument’s pose in the reference frame $R1_{0}$ (the reference frame at the base of the robotic arm R1 ). This is performed using R1 ’s wrist camera, which is calibrated to tune the distortion coefficients, along with its extrinsic and intrinsic parameters (OpenCV et al. 2024). The estimated STag pose is averaged using a Cartesian pose filter to remove jitter. Next, we calibrate the camera position on R1 to obtain the transform $T_{R1_{0}}^{\mathrm{cam}}$ .

The camera is rigidly attached to R1 . The transform $T_{R1_{0}}^{\mathrm{cam}}$ can be expressed through two distinct transformation pathways: one through the robot joints and one through the STag marker estimate. Each pathway has one unknown transform ( $T_{R1_{\mathrm{tool}}}^{\mathrm{cam}}$ and $T_{R1_{0}}^{\mathrm{STag}}$ , respectively), which are assumed to be fixed during the calibration process. We can solve for the two unknowns by minimizing the difference in the resultant $T_{R1_{0}}^{\mathrm{cam}}$ across multiple observations (Equation 1).

$$minimize\overset{i}{\underset{i=0:N-1}{\sum }}\left(T_{R1_{\mathrm{tool}}}^{\mathrm{cam}}\;T_{R1_{0},i}^{R1_{\mathrm{tool}}}-T_{\mathrm{STag,i}}^{\mathrm{cam}}\;T_{R1_{0}}^{\mathrm{STag}}\right).$$ (1)

We recover the desired transform with Equation 2:

$$T_{R1_{\mathrm{tool}}}^{\mathrm{cam}}\;=\;T_{R1_{0},i}^{\mathrm{cam}}\;{T_{R1_{0},i}^{R1_{\mathrm{tool}}}}^{-1}.$$ (2)

Here, the addition and subtraction signs are abstract representations of addition and subtraction in the SE(3) space, i.e., the manifold space. Additionally, the minimization problem can be addressed using an appropriate optimization tool. In this work, the equations were reorganized into a quadratic programming format, allowing the use of a solver from open-source libraries such as CVXPY or SciPy.

After estimating $T_{R1_{0}}^{\mathrm{cam}}$ , the detected STag markers can be described in the robot reference frame. Finally, the $STag_{xy}$ plane is forced to be parallel to the $world_{xy}$ plane, as per assumptions II and III.

During each localization run, R1 moves to the most recent STag pose, saved in R1 ’s memory, so the camera can localize the marker.

#### 2.4.3 Tactile detection of instruments

Given the approximate instrument’s pose using the STag marker, R1 now performs a more precise estimation using tactile detection directly on the instrument.

Considering Assumption II, any instrument can be used as a “reference” cube. Using ABB GoFa’s force-compliant motion (SoftMove), R1 physically touches the instrument on three orthogonal faces to identify its coordinate system (inst) . The touch points are defined with respect to STag ; it is recommended to use wider points to increase precision; however, we report that a spacing of 40 mm between co-planar points delivers sufficient performance.

Specifically, by using three points obtained from the same instrument’s face, we derive the first plane equation $\left(plane_{1}\right)$ . By projecting one of the two points from the second instrument’s face onto $plane_{1}$ , a third point is derived. Thus, it is possible to compute $plane_{2}$ . Repeating the same method with one point from the third instrument’s face, along with the information about $plane_{1}$ and $plane_{2}$ , we now have three nearly orthogonal planes (assuming that every set of three co-planar points is not collinear). Orthogonality is enforced by performing singular value decomposition (SVD) and setting the matrix of eigenvalues to the identity matrix.

Mathematically, let $p_{i,k}$ be point i on face k of the cube. Also, let $l_{ij,k}=p_{j,k}-p_{i,k}$ . We can get the normal to $plane_{1}$ as per Equation 3:

$$n_{1}=l_{12,1}\times l_{23,1},$$ (3)

and the equation of $plane_{1}$ as

$$n_{1}\cdot \left[\begin{array}{c} x\\ y\\ z \end{array}\right]=p_{1,1}.$$ (4)

To project one of the points on the second face onto $plane_{1}$ , we can use the following equation:

$$\left[\begin{array}{c} x\\ y\\ z \end{array}\right]=p_{1,2}+t\cdot n_{1}.$$ (5)

By solving Equation 4 using Equation 5, we obtain the third point $p_{3,2}$ to solve for $plane_{2}$ in the same way. The following steps can be repeated to obtain $p_{2,3}$ and $p_{3,3}$ for $plane_{3}$ .

Finally, we can stack the normal vectors to get $T=\left(\begin{array}{ccc} n_{1} & n_{2} & n_{3} \end{array}\right)$ . Then perform singular value decomposition (SVD) by solving Equations 6, 7.

$$U,S,V=SVD\left(T\right),$$ (6)

$$T_{R1_{0}}^{\mathrm{inst}}=U\;*\;V.$$ (7)

**FIGURE 4:**
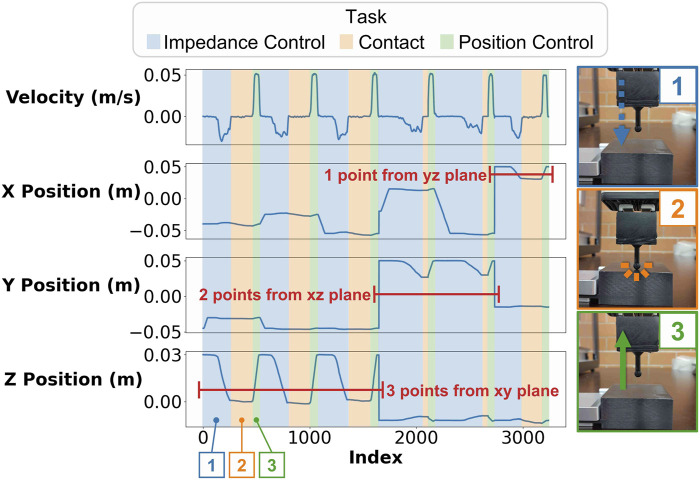
Plots showing the velocity and the position of the tool tip as it executes the touch feedback routine for six points in three perpendicular planes. The plots have different colors depending on the task the robot is executing (impedance control going down, contact, and position control going up). The contact point is estimated by considering when the tool’s velocity is 0 while R1 is in impedance control mode.

An example of the six-point touch feedback task is shown in Figure 4. The velocity graph of R1 has a negative value as the probe approaches the instrument face, and it reaches 0 when contact is made. This velocity profile is used to identify the position of the probe $\left(R1_{\mathrm{tool}}\right)$ when contact is made.

While performing the six-point touch feedback, the robot applied a negligible force to the instruments. Given that laboratory equipment typically weighs tens of kilograms and is equipped with high-friction rubber feet, no displacement was observed. However, if this method were to be applied to lighter instruments, there could be a risk of displacement due to the applied force.

#### 2.4.4 Defining the handover position

In the last step of the method, we obtain the location of the handover point, i.e., the point on each instrument where the plate must be picked and placed. Using Assumption I, we can derive $T_{R2_{0}}^{\mathrm{inst}}$ . inst origin is found at $plane_{1}\cap plane_{2}\cap plane_{3}$ , which is typically not coincident with the handover position reference frame (handover) . $T_{\mathrm{inst}}^{\mathrm{handover}}$ is a hardcoded and must be specified for each instrument.

By using $T_{R2_{0}}^{\mathrm{handover}}$ , R2 can transfer a plate to handover with a precision of ±1.2mm .

#### 2.4.5 Model generalizability

The framework presented in the previous sections is generalizable. Any instrument that follows assumptions II, III, and IV is suitable, considering some device-specific adjustments. To demonstrate this, we provide four implementation examples; one is represented using the mockup instruments, as shown in Figures 8, 10, and three are real instruments, as shown in Figure 11. We listed them in order of increasing adaptation difficulty:

• MockupInstrument : this represents the ideal case where the STag marker is attached to the right-front edge of a cube. The touch point locations on the three planes can be chosen without constraints. It should be noted that two distinct mockup instruments are used in this study; however, both share the same main features (a cube with a visible STag marker).

• PlateReader : the STag marker is attached on the right edge of the instrument, avoiding the slightly inclined surface at the front (to respect Assumption III). The touch points must avoid the mentioned tilted surface and the opening on the front-right of the instrument.

• PlateSealer : the STag is attached to the right-front edge of the instrument. Two touch point areas are wide, while the third plane (greyish stripes to the sides of the instrument’s screen) is quite small. However, R1 ’s tool can touch it.

• PipettingStation : the STag is attached to a 3D-printed support, not on the top surface. The instrument is tall, and the webcam needs some distance from the STag; this can cause R1 to reach the limit of its work envelope and stop. The instrument has no sharp edges, so the touch planes are created with a 3D-printed shell bolted on the instrument (front-right of the instrument). Touch points are chosen on this shell.

### 2.5 Experimental tests

To benchmark this combined approach, we compare three strategies for the placement of the microplates:

• “VL” uses a VL marker to achieve fine positioning, after which SIMO performs the placing task. To only estimate the precision of VL positioning, the handover pose is precisely hardcoded before running the test.

• “CV” uses the strategy described in Sections 2.4.2 and 2.4.4 to obtain the handover pose (the pose is not refined using the method described in Section 2.4.3). The strategy described in Section 2.4.4 is slightly modified in this case; from $T_{R1_{0}}^{\mathrm{STag}}$ , we can compute $T_{R2_{0}}^{\mathrm{STag}}$ , and by hardcoding $T_{\mathrm{STag}}^{\mathrm{handover}}$ , we can compute $T_{R2_{0}}^{{handover}^{\prime }}$ .

• “CV + TF” uses the strategy described in Sections 2.4.2 and 2.4.3 to refine the instrument’s pose. Then, it follows the procedure detailed in Section 2.4.4 to estimate $T_{R2_{0}}^{\mathrm{handover}}$ .

**FIGURE 5:**
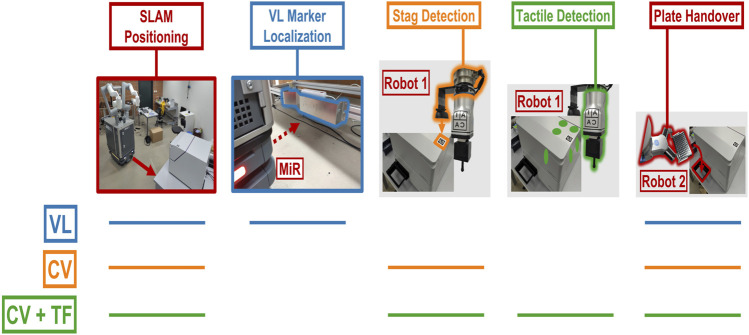
Overview of the strategies to perform the plate pick-and-place task. The colored lines indicate the specific actions performed in each strategy: “VL,” “CV,” and “CV + TF.”

All methods use SLAM for navigation, but only the VL strategy uses the “VL” marker for fine positioning. A visual summary of the three strategies is shown in Figure 5.

When experiments are repeated, SIMO retracts from the experimental station’s docking point, returns to the same position, and performs the placing task.

## 3 Experimental setup

### 3.1 Specialization of the grippers

R1 has a parallel gripper that holds a bespoke tool (Figure 2), which is used to touch the instruments while R1 is in impedance control mode. We designed the tool to have a cubic shape (50 × 50 × 40 mm) that can be easily gripped, with two inserts that mirror the shape of the gripper’s fingers. The tool’s probe is 30 mm long and has a spherical shape at the end with a diameter of 10 mm. The length of the tool’s stem allows it to reach and touch points on all planes, while the spherical tip ensures that there is a single point of contact between the tool and the touched surface. It should be noted that while the tool’s z-axis is parallel to the normal of $plane_{1}$ while touching it, the same axis is perpendicular to the normal of $plane_{2}$ and $plane_{3}$ (Figure 3) while performing the touch detection routine.

R2 mounts a finger gripper to hold the plates (Figure 2). We specialized the gripper by adding longer aluminum fingers and gluing silicone stripes at their ends, which provides sufficient grip to hold the plates. Finally, we also used spacers to enhance the gripper’s stroke and hold a plate in landscape mode.

### 3.2 System diagram topology

**FIGURE 6:**
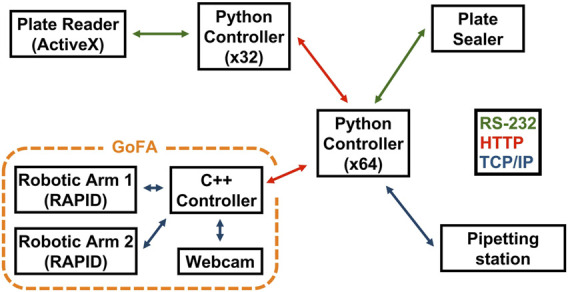
Overview of the hardware modules and their communication protocols; each node represents a module in the workflow. Interconnecting lines represented with different colors detail the communication protocols used between the various modules.

Figure 6 shows the system’s diagram. The central controller orchestrates the full protocol. It prompts SIMO to dock on a specific experimental station and requests to start device-specific action to the different instruments. However, such actions can be categorized into three main classes: “Receive Plate,” which prepares the instrument for plate placement; “Run,” which prompts the instrument to execute its specific task; and “Give Plate,” which sets the instrument to hand the plate back to the mobile robot.

### 3.3 Mockup instruments

Figures 8, 10 show the two types of mockup instruments that we used to compare the success rate and precision of various plate-placing strategies (see Sections 2.5 and 4.1). The first $\left(mockup_{1}\right)$ is comprised of a 3D-printed cube (87 × 95 × 42 mm) having a rectangular holder to fit an aluminum handover position. The handover position’s edges are chamfered to ease plate insertion, which is typical for automation-friendly instruments. An STag marker is placed on the top surface. The second $\left(mockup_{2}\right)$ has the same 3D-printed cube lying on a sandwich of a thin silicone layer (Dragon Skin 20, Smooth-On, USA) to ensure plate adhesion and a sheet of paper. For this experiment, both the cube and plate have an STag marker to compute their relative distance and evaluate placement precision through computer vision using R1 ’s wrist camera.

### 3.4 Room

**FIGURE 7:**
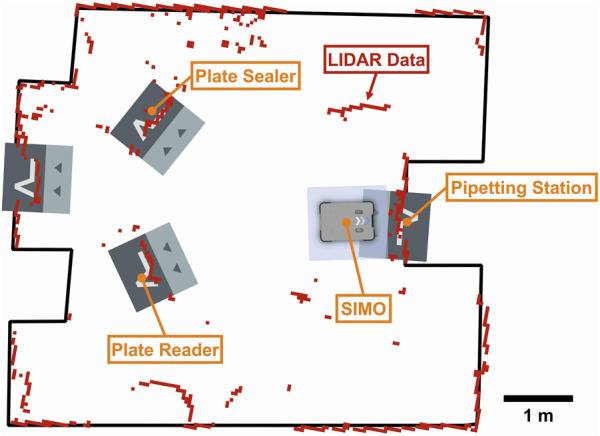
Map of the laboratory, generated by the MiR’s LiDAR, showing the positions of the three experimental stations: the plate sealer, plate reader, and pipetting station. The gray, double-colored squares represent recorded navigation points, while the robot’s current position is shown as a monocolored gray rectangle labeled “SIMO.” The red marks indicate real-time LiDAR data, with the black lines representing the room’s hardcoded layout. If the LiDAR data (red) does not align with the black lines—for example, showing red dots or lines in the room’s interior—this indicates that objects are obstructing the LiDAR sensor.

The laboratory map is generated using laser scanning (Figure 7). The highlighted shapes represent the docking stations where the VL markers are physically placed. Every VL marker is associated with an experimental station. We placed three experimental stations at the edges of the room, one for every instrument (pipetting station, plate sealer, and plate reader).

Typically, laboratory instruments are arranged along various walls, either close together on the same bench or on separate benches, sometimes at different angles to accommodate spacing needs. Although this setup can be challenging, it is not the most complex scenario, as other factors, such as obstacles or additional equipment, can add even greater complications.

## 4 Results

### 4.1 Mockup instrument test

Our method is initially validated by comparing it with different localization strategies using the two mockup instruments. The first experiment seeks to identify the success associated with placing a plate in the mockup instrument $\left(mockup_{1}\right)$ using the three methods described in Section 2.5.

**FIGURE 8:**
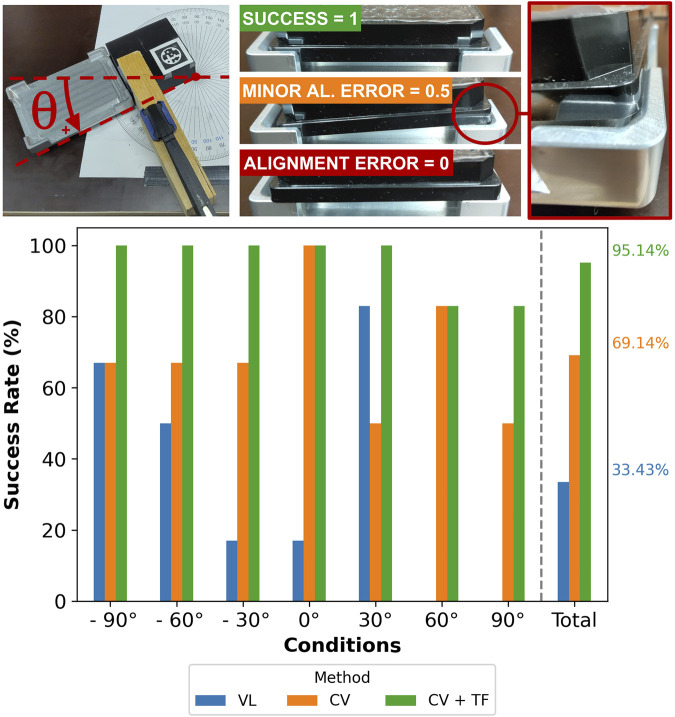
Assessment of the success rate for different methods (“VL,” “CV,” and “CV + TF”) and several angular displacements (ranging from −90° to +90° ) of the mockup instrument $\left(mockup_{1}\right)$ . The visual criteria to compute the success rate are also shown with photographs. The “CV + TF” method provides more consistent results among all conditions, followed by “CV” and “VL.” Every condition (defined by the angular displacement) was tested three times for each method.

Figure 8 shows $mockup_{1}$ . By using a protractor and a clamp, it was possible to rotate the cube while preventing undesired movements when running the tactile detection. We admit three possible outcomes: “success” (score=1) , “alignment error” (score=0) , and a “minor alignment error” (score=0.5) . A “minor alignment error” placement implies that the plate would fall into the “success” category with a slight movement or vibration. This is common for instruments that have retractable handover positions.

We tested the three methods (“VL,” “CV,” and “CV + TF”) by placing the mockup instrument in seven different orientations, ranging from −90° to +90° . Each test was repeated three times, yielding a total of 63 experiments (21 per method). As described in Section 2.5, SIMO uses SLAM to navigate to the instrument’s approximate position from which we assume that the robot can see the fiducial marker placed on $mockup_{1}$ .

**FIGURE 9:**
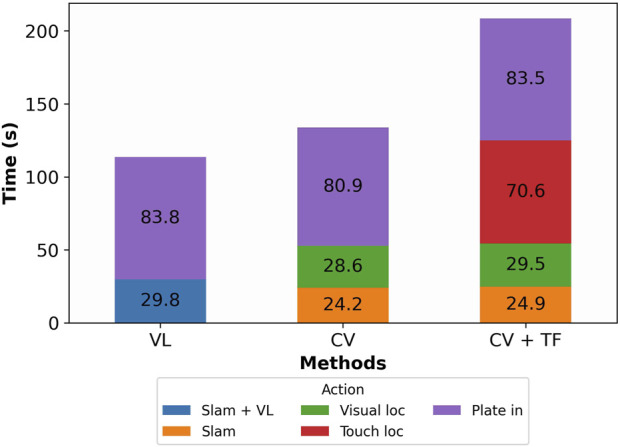
Column plot showing the total execution time for three methods (“VL,” “CV,” and “CV + TF”). The single column is split into colored rectangles that identify different actions performed by the robot; the time to run single actions is enclosed in the corresponding rectangle. “VL” is the fastest method, followed by “CV” and “CV + TF.”

Figures 8, 9 show that while “CV + TF” requires almost double the time compared to “VL,” its global success rate ( 95.14% over 21 placements in different orientations) justifies its adoption over the other methods. We also notice that the “VL” score would be close to 0% if the handover position was not hardcoded beforehand, highlighting the low robustness of this method. In addition, “CV” places in the middle of both metrics (success rate and time).

Analyzing the data from Figure 8, Table 1 confirms that at least one group’s mean is statistically different from the others (the ANOVA test). Additionally, the Tukey test shows that the means of all groups are statistically different.

**TABLE 1:** ANOVA and Tukey HSD test results.

Test	Result
ANOVA F-statistic	16.5372
ANOVA p-value	1.9038e-06

Group 1	Group 2	Mean difference (success rate)	p-adj	Reject
CV	CV + TF	0.2619	0.0477	True
CV	VL	−0.3571	0.0045	True
CV + TF	VL	−0.6190	0.0	True

**FIGURE 10:**
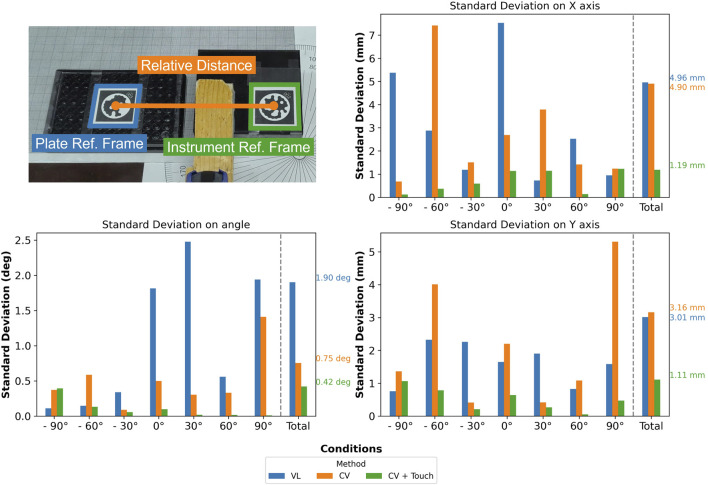
Assessment of the standard deviation (X, Y, and angular) of different methods (“VL,” “CV,” and “CV + TF”) for several angular displacements (ranging from −90° to +90° ) of the mockup instrument $\left(mockup_{2}\right)$ . The photograph on top details how the standard deviation is computed; the mockup instrument (in green) and the plate (in blue), respectively, have a fiducial marker that is used to compute their relative pose and its associated standard deviation.

In the second experiment, we use $mockup_{2}$ . The goal is to link the success rate with a quantitative metric: the relative difference between the plate reference frame and a fixed frame, named “instrument reference frame” (see Figure 10). By focusing on the relative distance’s standard deviation rather than its absolute value, we can obtain the placing precision of the different methods based on three metrics: X, Y, and angular standard deviations.

As in the previous experiment, by using a protractor and a clamp, it was possible to rotate the cube while preventing undesired movements when running the tactile detection; we also tested the same number of conditions to yield 63 experiments in total (21 per method).

From Figure 10, it is noticeable that “CV + TF” consistently outperforms the other strategies, explaining the higher success rate obtained, as shown in Figure 8. Additionally, we hypothesize that “CV’s” lower angular standard deviation, if compared to “VL,” determines the doubling of its success rate since the X and Y standard deviations are comparable.

In conclusion, “CV + TF” delivers the best performance, achieving a precision of ±1.2 mm and ±0.42° , although with a longer processing time. As such, it will be the default method used in the following sections. Additionally, in the following sections, “CV + TF” incorporates the VL marker localization step to further improve the method’s overall robustness.

### 4.2 Real instrument tests

Following the mockup instrument tests, we next evaluate the performance of real instruments that have varied geometries and placement types. By doing this, we want to test the method’s generalizability, first introduced in Section 2.4.5.

**FIGURE 11:**
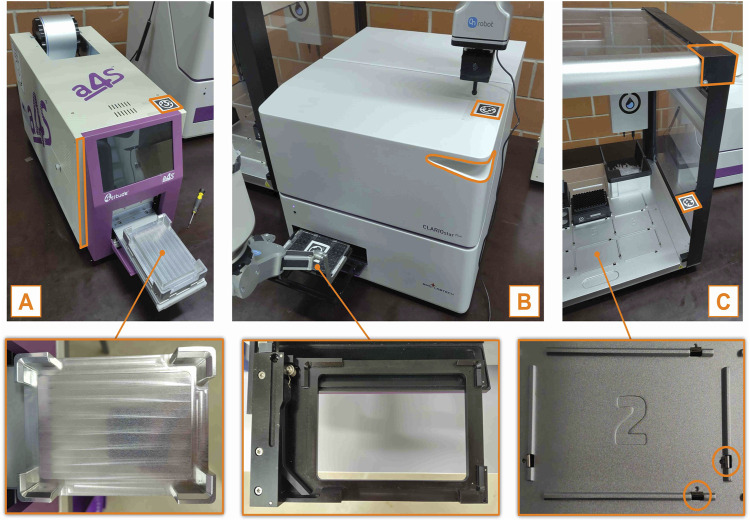
Photographs that show the three real instruments used to test the “CV + TF” method. **(A)**: Plate sealer, **(B)**: Plate reader, **(C)**: Pipetting station. The important implementation details are highlighted in orange, while the photographs in the second row show the instruments’ handover position.

Figure 11 illustrates the tested instruments, in order of increasing pick-and-place difficulty, from left to right. Each instrument was tested 10 times adding random noise each time: ±10 mm and ±5° . The instrument was re-positioned to its default position ( $handover_{yz}\|world_{yz}$ , relativedistance=50 cm ) after every test, before re-adding noise.

The plate sealer’s handover position has sharp, chamfered edges (Figure 11A) that facilitate plate placement, resulting in a 100% success rate during testing. In contrast, the plate reader’s handover position is less robust, with shorter edges and a smaller chamfer (Figure 11B). Nevertheless, we report a 100% success rate.

The pipetting station presents a more significant challenge for pick-and-place operations. As shown in Figure 11C, the station features very small chamfered edges and small metal lips that guide the plate into position. Although this design simplifies plate insertion for humans, it demands millimeter-level precision from robots to avoid failure. The flexibility of this part allows humans to easily adapt through learned behavior, while robots, which rely more on precise control and have less sophisticated adaptive feedback, are more likely to fail the task. Although this metal part is removable, we decided to test SIMO in a more challenging scenario; we report a 100% success rate.

### 4.3 Large-scale stress test

Finally, we use SIMO to run an experiment that requires all three instruments mentioned in the last section, CMC determination, to test the method’s robustness over extended periods of time. We defined this to be the “large-scale stress test.” The room was organized as per Section 3.4.

**FIGURE 12:**
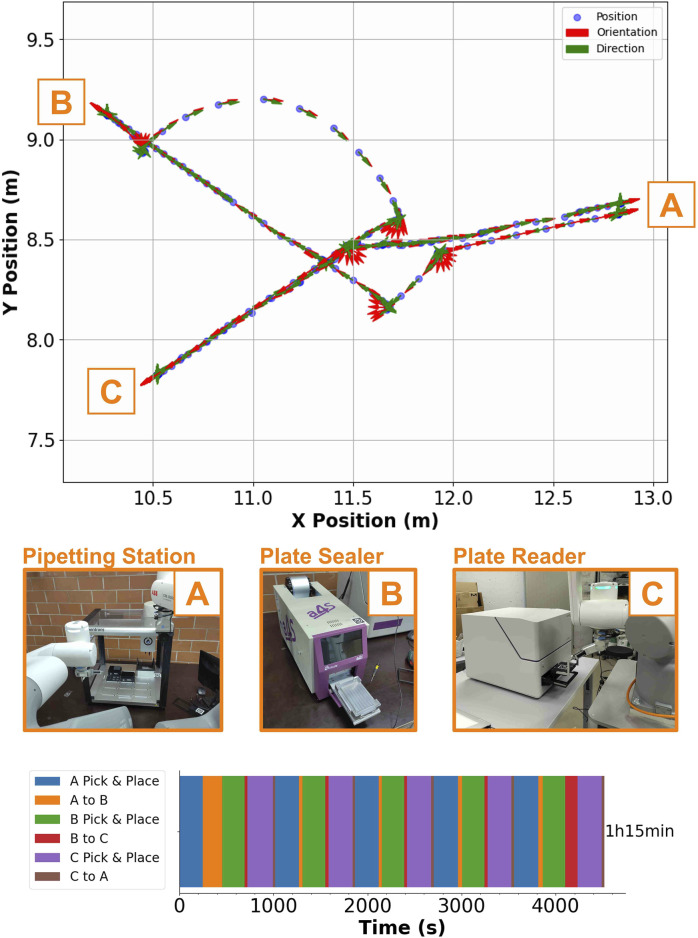
Summary of the large-scale stress test. The upper plot shows the robot’s position in the room as it is executing one round of the CMC experiment; the orange letters highlight the positions of the three experimental stations on the map (**(A)**: Plate sealer, **(B)**: Plate reader, **(C)**: Pipetting station). The photographs in the middle illustrate the robot–instrument interaction, while the single horizontal column plot in the lower part shows the total time to run the CMC protocol five times in a row (**(A)**: Plate sealer, **(B)**: Plate reader, **(C)**: Pipetting station). Every color highlights the time to perform a specific task to complete the experiment.

We report that SIMO can run the CMC experiment five times in a row without failure (total time of experiment = 1 h 15 min). Figure 12 shows the robot’s trajectory over one run; after placing the plate in the pipetting station, SIMO brings the standard microplate filled with the reagents to the plate sealer, which applies a plastic cover to the plate to avoid evaporation. Finally, SIMO brings the standard microplate to the plate reader, where the fluorescence signal from the experiment is read; the raw data can be processed to extract the CMC. The analyzed plate is discarded, and a new cycle can start. This consistent performance across multiple cycles underscores the robustness and reliability of our approach.

## 5 Conclusion

In this work, we present an algorithm that couples visual and tactile feedback to achieve fine pick and place ( ±1.2mm and ±0.4° ) of standard microplates. We tested this method on a bi-manual mobile robot, named SIMO, to interact with different laboratory instruments. This approach was validated on mockup instruments by comparing its performance against two other common strategies. Subsequently, we demonstrated the method’s ease of use and robustness by adapting it to three real instruments. Finally, we showed the method’s robustness over extended periods by executing the CMC determination protocol five times, for a total experiment time of 1 h 15 min.

Future improvements to our method might include generalizing it to multiple mobile manipulators, designing a single compact gripper capable of vision and touch perception coupled with pick-and-place capabilities, and developing error recovery strategies. For instance, if a marker is not detected by the camera, investigating possible fiducial marker search strategies could be beneficial. Although the presented method demonstrates significant promise, certain bottlenecks may limit its overall throughput and efficiency. One of the primary challenges is represented by the sequential nature of robotic tasks, where the robot must complete one step before advancing to the next. This leads to potential downtime, especially in high-throughput environments, requiring the simultaneous handling of multiple tasks. Additionally, interactions with instruments, such as calibration and localization using fiducial markers, can become time-intensive if environmental conditions change or markers are not promptly detected. To overcome these limitations, future work could focus on enabling parallel task execution with multiple robots, optimizing path planning algorithms to minimize idle time, and advancing sensor fusion techniques to enhance localization speed and accuracy. Finally, it would also be useful to test the wet laboratory experimental robustness of the system by performing real chemistry and biology experiments to assess which additional benefits a robotic platform can bring to the laboratory. These enhancements could significantly boost throughput and fully harness the potential of mobile robotic systems in dynamic laboratory environments.

Efforts to achieve universal, reliable robotic localization in dynamic environments have the potential to reshape wet laboratory research. Automating non-value-adding activities such as labware transportation will improve the reliability and reproducibility of experimental data by virtually eliminating human error.

Human error in laboratory settings can manifest in various ways, such as fatigue, distraction, or minor inconsistencies in manual dexterity, which introduce variability into experimental procedures. Manual data recording errors, such as incorrect measurements or conditions, further compound inaccuracies. Automating repetitive, precision-critical tasks, including pipetting or plate handling, allows for consistent, millimeter-level accuracy and error-free data collection. This can greatly benefit routine protocols like CMC, enabling a new experimental pace with mobile robots that can potentially work continuously, unlike humans.

## Data Availability

The original contributions presented in the study are included in the article/supplementary material; further inquiries can be directed to the corresponding author.
